# Genome-wide Twist1 occupancy in endocardial cushion cells, embryonic limb buds, and peripheral nerve sheath tumor cells

**DOI:** 10.1186/1471-2164-15-821

**Published:** 2014-09-28

**Authors:** Mary P Lee, Nancy Ratner, Katherine E Yutzey

**Affiliations:** Division of Molecular Cardiovascular Biology, Cincinnati Children’s Hospital Medical Center, Cincinnati, Ohio 45229 USA; Division of Experimental Hematology and Cancer Biology, Cincinnati Children’s Hospital Medical Center, Cincinnati, Ohio 45229 USA

**Keywords:** Twist1, Endocardial cushions, Limb buds, Peripheral neuron sheath tumor cells, ChIP, ChIP-seq

## Abstract

**Background:**

The basic helix-loop-helix transcription factor Twist1 has well-documented roles in progenitor populations of the developing embryo, including endocardial cushions (ECC) and limb buds, and also in cancer. Whether Twist1 regulates the same transcriptional targets in different tissue types is largely unknown.

**Results:**

The tissue-specificity of Twist1 genomic occupancy was examined in mouse ECCs, limb buds, and peripheral nerve sheath tumor (PNST) cells using chromatin immunoprecipitation followed by sequencing (Chip-seq) analysis. Consistent with known Twist1 functions during development and in cancer cells, Twist1-DNA binding regions associated with genes related to cell migration and adhesion were detected in all three tissues. However, the vast majority of Twist1 binding regions were specific to individual tissue types. Thus, while Twist1 has similar functions in ECCs, limb buds, and PNST cells, the specific genomic sequences occupied by Twist1 were different depending on cellular context. Subgroups of shared genes, also predominantly related to cell adhesion and migration, were identified in pairwise comparisons of ECC, limb buds and PNST cells. Twist1 genomic occupancy was detected for six binding regions in all tissue types, and Twist1-binding sequences associated with *Chst11*, *Litaf*, *Ror2,* and *Spata5* also bound the potential Twist1 cofactor RREB1. Pathway analysis of the genes associated with Twist1 binding suggests that Twist1 may regulate genes associated with the Wnt signaling pathway in ECCs and limb buds.

**Conclusions:**

Together, these data indicate that Twist1 interacts with genes that regulate adhesion and migration in different tissues, potentially through distinct sets of target genes. In addition, there is a small subset of genes occupied by Twist1 in all three tissues that may represent a core group of Twist1 target genes in multiple cell types.

**Electronic supplementary material:**

The online version of this article (doi:10.1186/1471-2164-15-821) contains supplementary material, which is available to authorized users.

## Background

Twist1 is a highly conserved basic-helix-loop-helix (bHLH) transcription factor, which regulates cell migration, proliferation, and differentiation in progenitor populations, such as embryonic neural crest cells, cranial mesoderm, limb buds, and endocardial cushions (ECCs) [1–4]. In addition to its role in developmental processes, human TWIST1 is highly expressed in diseased heart valves and in metastatic tumor cells [5–7]. Twist1 expression is normally silenced after embryonic development, but expression is reactivated during disease, including cancer. Twist1 controls gene expression through binding E-box (5’-CANNTG-3’) consensus sites as a homodimer or heterodimer with other bHLH transcription factors [4, 8]. However, a comparison of Twist1 direct transcriptional targets in developing and diseased tissues has yet to be reported. This study focuses on identifying and comparing Twist1 genomic occupancy in developing ECC, limb buds, and a cancer cell line.

In multiple structures in the developing embryo, including the heart, limb buds, cranial sutures, skeletal elements, and neural crest derivatives, Twist1 regulates cell migration and proliferation of progenitor populations [4]. In the developing heart, *Twist1* expression is robust in the mesenchymal cells of the ECC and is subsequently down-regulated during remodeling and differentiation of the valve leaflets [9, 10]. In the ECC, Twist1 promotes cell migration and proliferation of mesenchymal cells. Direct transcriptional targets of Twist1 include genes involved in cell migration (*Tbx20, Cdh11, Sema3C*) and proliferation (*Rab39b* and *Gadd45a*) [2, 5, 9]. In the developing limbs, Twist1 is broadly expressed in the limb bud mesenchyme and strictly regulated genetic dosage of Twist1 is required for limb bud outgrowth and patterning [1, 3, 11]. Although extensive genetic studies have been performed demonstrating an essential role for Twist1 in limb patterning and morphogenesis, the direct transcriptional targets underlying these functions are only recently being elucidated [12]. In addition, it is not known if Twist1 regulates the same downstream targets in distinct progenitor populations or if there is tissue-specificity to Twist1 regulatory mechanisms in the developing embryo.

While Twist1 is largely absent from adult differentiated tissues, it is expressed in diseased heart valves and highly metastatic cancers such as breast, pancreatic, gastric, prostate, and malignant peripheral nerve sheath tumors (MPNST) [6, 7, 13, 14]. Human MPNST cells can harbor *NF1* and *p53* mutations, and murine *NF1* and *p53* mutations can cause similar nerve-associated sarcomas, peripheral nerve sheath tumors (PNST) [14–16]. siRNA-mediated knockdown of *Twist1* abrogates the migratory activity of human MPNST cells *in vitro* [14, 16]. Twist1 also is expressed in invasive carcinoma and promotes endothelial-to-mesenchymal transformation (EMT) and metastasis of tumor cells through regulation of genes involved in differentiation, adhesion, and proliferation [6, 17]. It has not previously been determined if the downstream targets of Twist1 related to regulation of cell migration or proliferation in embryonic structures and cancer cells are shared or are cell-type specific.

To investigate the tissue-specificity of Twist1 genomic occupancy, we performed chromatin immunoprecipitation (ChIP) with anti-Twist1 followed by NextGen Sequencing (ChIP-seq) in mouse embryonic day (E)12.5 ECCs, E10.5 forelimb buds, and cultured PNST cells. The specificity of Twist1 binding was confirmed by ChIP qPCR in each tissue. Potential Twist1 cofactors and intersecting signaling pathways also were identified.

## Results

### Analysis of Twist1 genomic occupancy in ECCs, limb buds, and PNST cells

**Figure 1:**
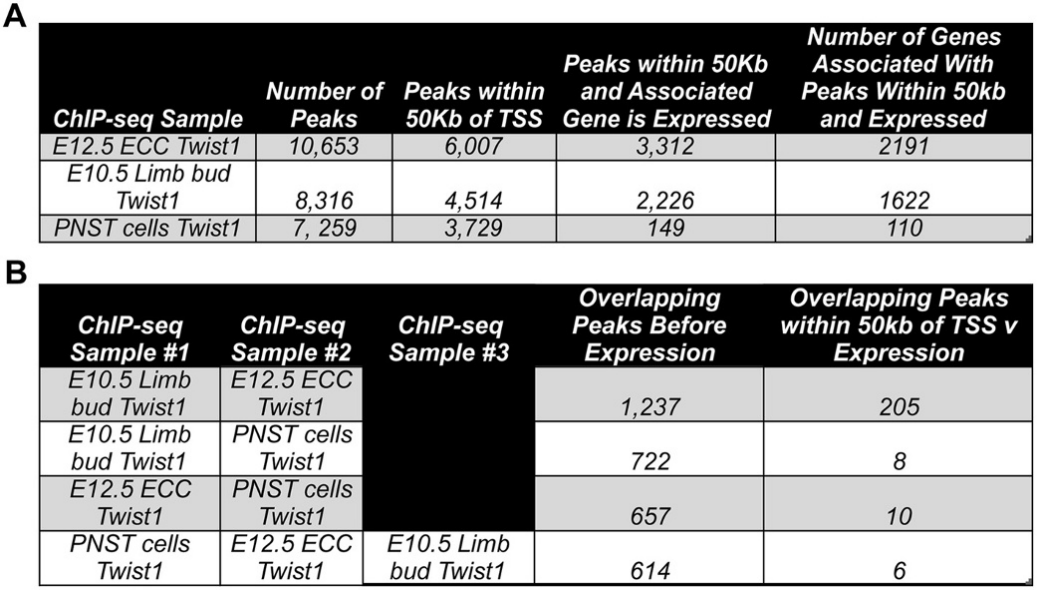
**Twist1 genomic occupancy in endocardial cushions, limb buds, and peripheral nerve sheath tumor cells. A**. Twist1 binding regions were identified by ChIP-seq followed by MACS analysis in E12.5 ECCs, E10.5 limb buds, and PNST cells compared to a control strain matched whole genome dataset. The total number of peaks, the number of peaks within 50 kilobases (Kb) of a transcriptional start site (TSS), and the number of peaks associated with expressed genes, as determined by microarray gene expression analysis of the tissue of interest, are shown. **B**. The total number of shared peaks detected by Twist1 ChIP-seq of ECC-Limb, Limb-PNST, ECC-PNST and ECC-Limb-PNST groups is shown. The number of overlapping peaks within 50 kb of a TSS for expressed genes also was determined.

Twist1 genomic occupancy was assessed by ChIP with anti-Twist1 in mouse E12.5 ECCs, E10.5 limb buds, and PNST cells followed by deep sequencing (ChIP-seq). Next Gen Sequencing was used to generate 3,651,572 reads for E12.5 ECCs, 3,282,621 reads for E10.5 limb buds, and 9,918,784 reads for PNST cells. Resulting sequences were aligned to the mouse genome (mm9), and peaks were discovered using MACS analysis. A p-value cut-off of 1.00e-05 was used to identify 10,653 peaks for E12.5 ECCs, 8,316 peaks for E10.5 limb buds, and 7,259 peaks for PNST cells (Figure 1A, Additional files 1, 2 and 3) relative to comparable reads for FVB/N mouse genomic DNA as a control. In order to confirm enrichment of E-box binding sites in each data set, *de-novo* motif enrichment was performed using PscanChIP and MEME-ChIP (Additional file 4) [18, 19]. E-box containing motifs were significantly enriched in all datasets when analyzed by PscanChIP and MEME-ChIP (Additional file 4). Peaks were filtered based on distance from the nearest transcriptional start site (TSS) with a threshold of 50 kilobases (Kb) resulting in 6,007 peaks for E12.5 ECCs, 4,514 peaks for E10.5 limb buds, and 3,729 peaks for PNST cells (Figure 1A). In order to compare Twist1 genomic occupancy with genes expressed in each tissue, genes associated with binding peaks in each tissue were compared to respective Affymetrix microarray gene expression data for E12.5 ECCs, E10.5 limb buds (forelimbs), and PNST cells [10, 16, 20]. The identities of genes associated with binding peaks within 50 Kb of TSS, identified by ChIP-seq were compared to the total expressed genes as detected by microarray for each tissue individually (Figure 1A, Additional file 5: Figure S5). These comparisons were used to generate lists of Twist1 binding regions corresponding to expressed genes in each tissue. For E12.5 ECCS, 3,312 Twist1 binding peaks corresponding to 2191 expressed genes were identified (Figure 1A). Twist1 occupancy was detected for 2,226 peaks corresponding to 1622 expressed genes in E10.5 limb buds, and 149 peaks corresponding to 110 expressed genes were detected for PNST cells (Figure 1A). The list of genes for PNST cells is likely an underestimate of Twist1 genomic occupancy because only genes that are expressed in both human MPNST and mouse PNST cell lines were included [14, 16]. These ChIP-seq data indicate that Twist1 occupies genomic regions associated with large numbers of genes expressed in ECCs, limb buds, and PNST cells.

Tissue-specific and shared gene targets of Twist1 in ECCs, limb buds, and PNST cells corresponding to binding regions within 50 Kb of TSS were analyzed [21]. Binding region overlap determination was performed before and after peaks were compared to gene expression profiles (Figure 1B). These analyses produced datasets containing binding regions that are unique to a specific tissue type or shared among multiple tissue types. Groups of binding regions were classified as ECC only, Limb only, PNST only, ECC-Limb, ECC-PNST, Limb-PNST, or ECC-Limb-PNST. E12.5 ECCs and E10.5 limb buds share the most binding regions, with 1,237 shared peaks in the ECC-Limb group before comparison to mRNA expression data, and 205 ECC-Limb binding regions associated with expressed genes after comparison with mRNA expression. Similarly the Limb-PNST group contains 722 shared binding regions, before the expression filter, with only 8 shared binding regions corresponding to expressed genes. The ECC-PNST group contains 657 total shared binding regions, with 10 shared binding regions corresponding to expressed genes. Surprisingly, only 6 binding regions associated with expressed genes were occupied by Twist1 in all three cell types (ECC-Limb-PNST), indicating a high degree of tissue-specificity in Twist1 genomic occupancy. Genomic sequences of the shared binding regions associated with expressed genes were analyzed for presence of an E-box consensus site on the sense or antisense strand (5’-CANNTG-3’), which would indicate a potential Twist1 binding site. Of the 205 ECC-Limb shared binding regions associated with expressed genes, 138 contain an E-box consensus site, corresponding to 108 genes. In addition, E-box consensus sites are present in 7/10 ECC-PNST shared binding regions, 6/8 of the Limb-PNST binding regions, and 5/6 of the ECC-Limb-PNST shared binding regions. Collectively, these ChIP-seq experiments demonstrate extensive Twist1 binding to genomic sequences and indicate that Twist1 bound predominantly in a tissue-specific manner, with much smaller numbers of shared binding regions detected among ECCs, limb buds, and PNST cells.

In order to examine Twist1-DNA binding distribution across the genome and determine if Twist1 is bound to specific genomic regions relative to gene organization, location analysis was performed. Twist1 binding regions located within 50 Kb of a TSS for genes expressed in each tissue were subjected to genomic location and peak distribution analysis using Genomatix RegionMiner (Additional file 6). Twist1 binding regions associated with expressed genes were analyzed in tissue-specific gene groups (ECC only, Limb only, and PNST only), and also in groups of binding regions shared between tissue types (ECC-Limb, ECC-PNST, and Limb-PNST). Distribution of the tissue-specific binding regions and ECC-Limb shared peaks is largely within intergenic and intronic regions. However, ECC-PNST and Limb-PNST shared binding regions are more evenly distributed in genomic regions. This lack of specificity in peak distribution relative to gene organization is consistent with other transcription factors, including Twist1, in other cell types [22, 23]. These data suggest that Twist1 occupies genomic sequences associated with expressed genes located in intergenic and intragenic genomic regions and does not bind exclusively to promoter regions.

### Functional classification of genes associated with Twist1 binding peaks

**Figure 2:**
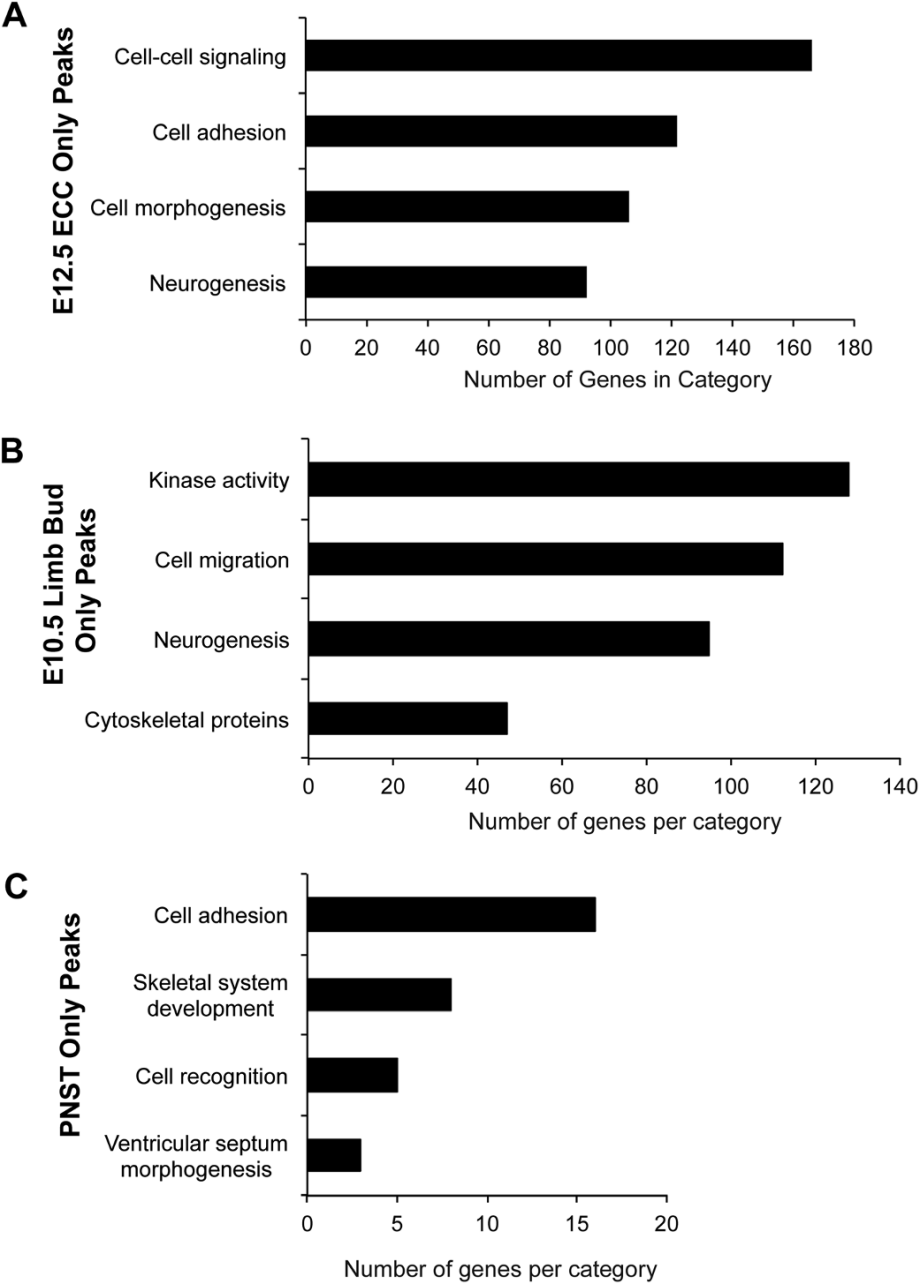
**Gene ontology analysis of the genes associated with Twist1 binding regions in ECCs, limb buds, and PNST cells. A, B, C**. ToppGene was utilized to perform GO analysis of the genes associated with Twist1 ChIP-seq peaks that are within 50 Kb of a transcriptional start site (TSS) of expressed genes in E12.5 ECCs (2191 genes), E10.5 limb buds (1622 genes), or PNST cells (110 genes). The four most predominant GO categories and the total number of genes in each category are represented.

The major biological functions of genes with Twist1 binding regions identified within 50 Kb of TSS, that also are expressed in ECCs, limb buds, or PNST cells, were assessed using gene ontology (GO) analysis (Figure 2). The four GO categories containing the greatest numbers of genes in each tissue were chosen for further analysis. In ECCs, the four predominant GO groups were cell-cell signaling, cell adhesion, cell morphogenesis, and neurogenesis (Figure 2A, Additional file 7). The top four GO categories of Twist1 occupied genes in limb buds were kinase activity, cell migration, neurogenesis, and cytoskeletal proteins (Figure 2B, Additional file 8). For PNST cells, the four GO groups with the most genes were cell adhesion, skeletal system development, cell recognition, and ventricular septum morphogenesis (Figure 2C, Additional file 9). Cell adhesion-cell migration was among the most predominant GO categories in each tissue type, indicating a conserved role for Twist1 in cell adhesion-migration in multiple cellular contexts (Figure 2A,B,C). However, the specific genes corresponding to Twist1 binding regions in each of these GO categories were unique to each tissue type, suggesting cell-type specificity of Twist1 genomic occupancy of gene targets associated with cell adhesion and migration.

**Figure 3:**
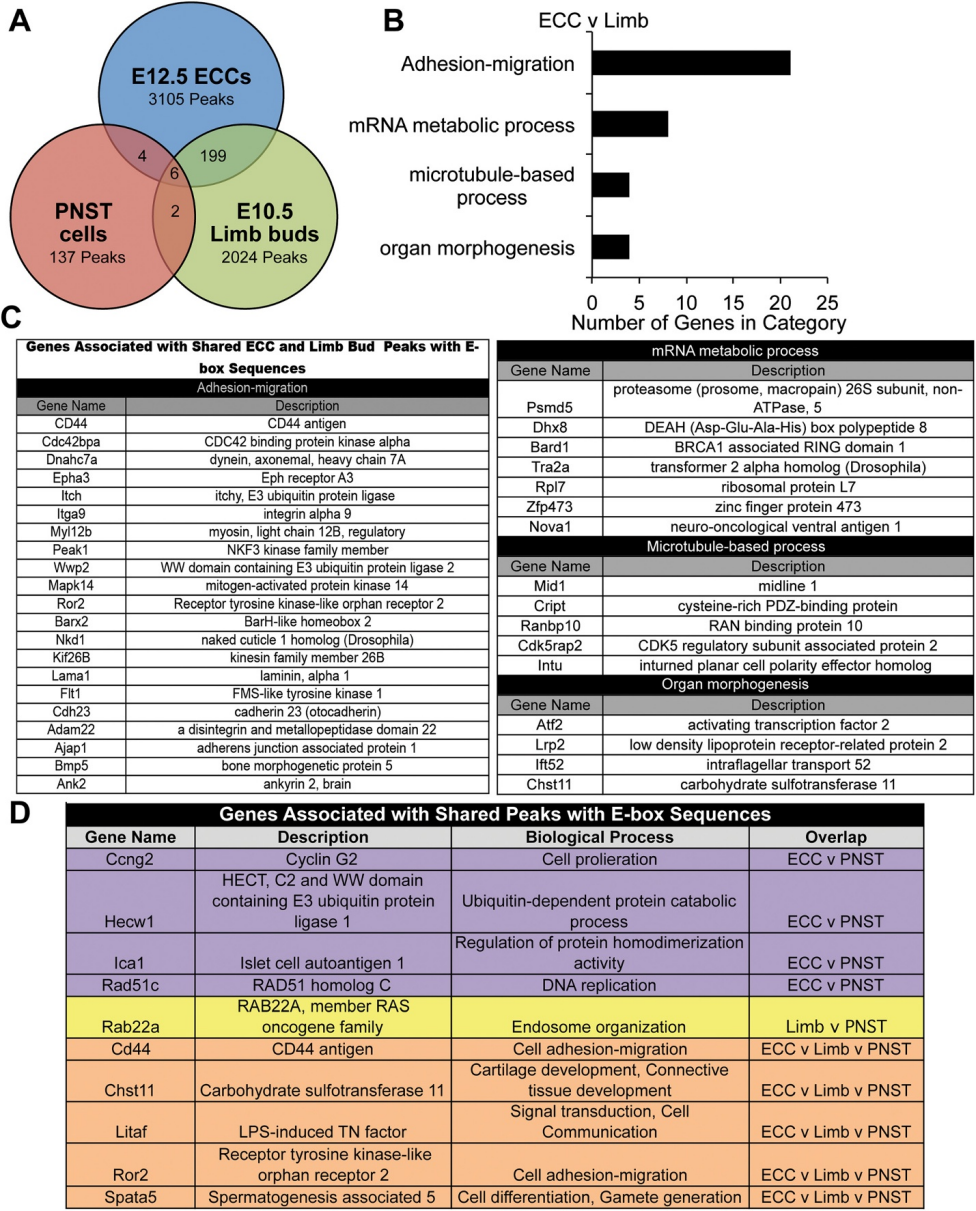
**Shared Twist1 binding regions detected by ChIP-seq in ECC, limb buds, and PNST cells. A**. Twist1 ChIP-seq peaks identified in E12.5 ECCs, E10.5 limb buds, and PNST cells were filtered based on a cut off of 50 Kb from the nearest TSS and gene expression as determined by microarray in each cell type. The peaks associated with expressed genes from each tissue type were analyzed for shared peaks and are represented in the Venn diagram. **B**. A total of 205 Twist1 binding regions were detected in both ECC and limb bud, and 138 contain E-box consensus sequences. The four predominant GO categories for the 108 genes associated with shared ECC-Limb E-box-containing binding regions are shown. **C**. Gene names and descriptions of the four GO categories containing the most genes for ECC-Limb E-box containing binding regions from B are shown. **D**. Gene names and descriptions are shown for shared binding regions detected in ECC-PNST, Limb-PNST, and ECC-Limb-PNST groups.

The biological functions of ECC-Limb shared binding regions associated with expressed genes were further examined resulting in 108 genes associated with the 138 binding regions containing an E-box consensus site (Figure 3B and C). Again, adhesion-migration was the most predominant GO category in genes associated with ECC-Limb shared binding regions containing E-box consensus sites associated with expressed genes. Additional GO categories include mRNA metabolic process, microtubule-based process, and organ morphogenesis. Thus, the expressed gene identities associated with Twist1 shared binding regions in ECCs and limb buds suggest conserved functions for Twist1 in regulation of cell adhesion-migration during development in different tissues. Expressed genes associated with shared binding regions containing E-box binding sequences related to cell adhesion and migration also were identified for ECC-PNST, Limb-PSNT and ECC-Limb-PNST groups (Figure 3D). The overlapping peaks in the ECC-Limb-PNST group correspond to six shared binding regions associated with 5 genes: *CD44 antigen (Cd44), carbohydrate sulfotransferase 11 (Chst11), LPS-induced TN factor (Litaf), receptor tyrosine kinase-like orphan receptor 2 (Ror2),* and *spermatogenesis associated 5 (Spata5)*. These five genes relate to the biological processes of cell adhesion-migration, cell differentiation, signal transduction, and cartilage development (Figure 3D). These biological processes are consistent with known Twist1 functions during embryogenesis and in cancer cells.

### Validation of Twist1 genomic occupancy in ECC, limb buds and PNST cells

**Figure 4:**
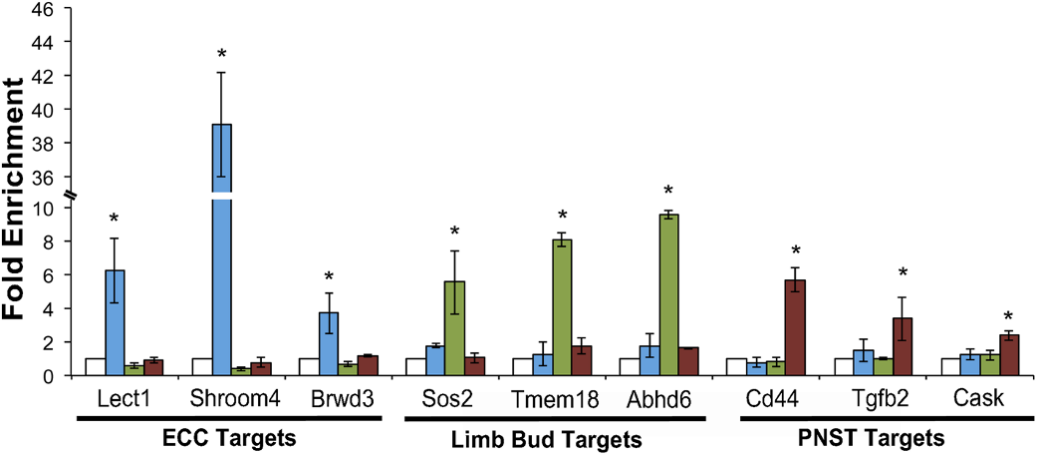
**Validation of Twist1 ChIP-seq peak specificity ECC, limb, or PNST cells.** ChIP was performed with anti-Twist1 in E12.5 ECCs (blue bars), E10.5 limb buds (green bars), and PNST cells (red bars) followed by sequence-specific qPCR to examine relative binding of Twist1 to the three binding regions with the highest fold enrichment, from MACS analysis, associated with expressed genes in each tissue. In E12.5 ECCs, Twist1 occupies binding regions associated with *leukocyte cell derived chemotaxin 1* *(Lect1), shroom family member 4 (Shroom4)*, and *bromodomain and WD repeat domain containing 3* *(Brwd3).* In E10.5 limb buds, Twist1 occupies binding regions associated with *son of sevenless homolog 2* *(Sos2), transmembrane protein 18 (Tmem18),* and *abhydrolase domain containing 6 (Abhd6)*. In PNST cells, Twist1 occupies binding regions associated with *CD44 antigen (Cd44)* and *calcium/calmodulin-dependent serine protein kinase (Cask)*, and *transforming growth factor, beta 2 (Tgfb2)*. ChIP quantification was performed by qPCR, and fold-enrichment was calculated versus an anti-rabbit IgG control for each tissue. All analyses were performed in biological triplicate. * indicates p < 0.05 as determined by student’s *t*-test comparing IgG and Twist1 ChIP qPCR ΔΔCt values.

To further examine tissue-specificity of Twist1 binding, the top three E-box containing binding regions associated with expressed genes, as determined by the highest fold-enrichment in each cell type, were chosen for further analysis. ChIP with anti-Twist1 was performed in E12.5 ECCs, E10.5 limb buds, and PNST cells followed by quantification using qPCR relative to normal rabbit IgG control. ECC tissue-specific binding regions chosen for further analysis were associated with the genes *leukocyte cell derived chemotaxin 1(Lect1), shroom family member 4 (Shroom4)*, and *bromodomain and WD repeat domain containing 3(Brwd3)*. Twist1 ChIP followed by qPCR demonstrated binding of Twist1 to ECC-specific regions in ECCs, but not in limb buds or PNST cells (Figure 4). Likewise, the limb bud-specific binding regions were associated with the genes *son of sevenless homolog 2(Sos2), transmembrane protein 18 (tmem18),* and *abhydrolase domain containing 6 (abhd6),* and were bound by Twist1 in limb buds, but not in ECCs or PNST cells (Figure 4). Finally, PNST cell-specific binding regions associated with the genes *CD44 antigen (Cd44), calcium/calmodulin-dependent serine protein kinase (Cask)*, and *transforming growth factor, beta 2 (Tgfb2)* were bound by Twist1 in PNST cells, but not in ECC or limb buds (Figure 4). These data further support the tissue-specificity of Twist1 binding to genomic sequences detected by ChIP-Seq in ECC, Limb buds, and PNST cells.

**Figure 5:**
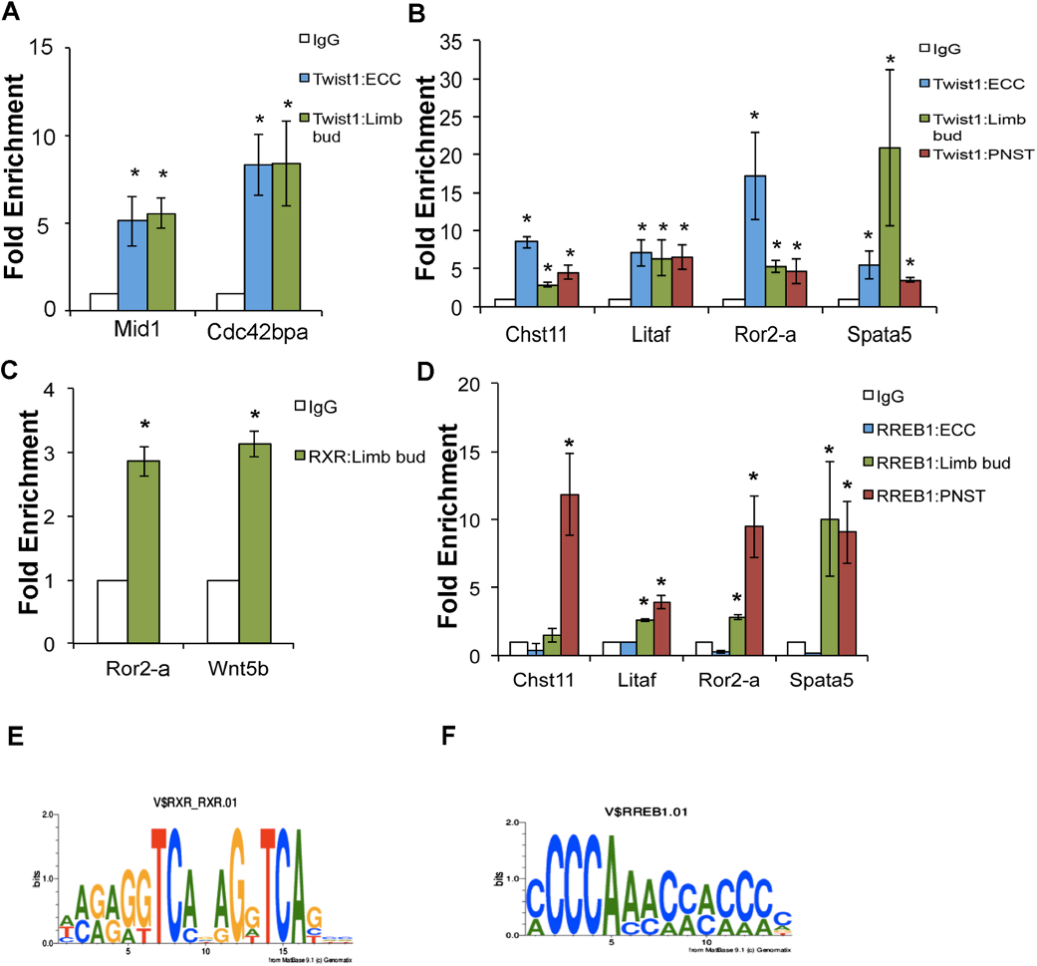
**Twist1 and candidate cofactors occupy binding regions shared among ECC, limb bud, and PNST cells. A**. Binding of Twist1 to regions associated with the genes *midline 1 (Mid1)* and *Cdc42 binding protein kinase alpha (Cdc42bpa)* detected in E12.5 ECCs and E10.5 limb ChIP-seq was validated by ChIP with anti-Twist1 in E12.5 ECCs and E10.5 limb buds followed by sequence-specific qPCR. **B**. Twist1 binding regions containing an E-box sequence, as detected by ChIP-seq in E12.5 ECCs, E10.5 limb buds, and PNST, are associated with *receptor tyrosine kinase-like orphan receptor 2 (Ror2-a), carbohydrate sulfotransferase 11 (Chst11), spermatogenesis associated 5 (Spata5)* and *LPS-induced TN factor (Litaf).* Twist1 ChIP followed by qPCR indicates that Twist1 binds each of these sequences in E12.5 ECCs, E10.5 limb buds, and PNST cells. **C**. Genomatix RegionMiner was utilized to identify RXR as a potential cofactor with Twist1 in E10.5 limb bud. Twist1 binding regions containing an E-box within 40 bp of an RXR binding consensus associated with *Ror2-a* and *Wnt5b* genes involved in limb development were chosen for validation*.* ChIP was performed with anti-RXR in E10.5 limb buds followed qPCR amplification of Twist1 binding region sequences. **D**. RREB1 consensus binding sites were identified within 40 base pairs of an E-box in Twist1 binding peak sequences of *Chst11, Litaf, Ror2-a*, and *Spata5* detected in ECC, limb bud and PNST cells. ChIP was performed with anti-RREB1 followed by qPCR amplification of Twist1 binding region sequences in ECCs, limb buds, and PNST cells. **E**. Motif of RXR consensus site. **F**. Motif of RREB consensus site. Fold-enrichment was calculated relative to normal rabbit IgG control. All analyses were performed in biological triplicate. * indicates p < 0.05 as determined by student’s *t*-test comparing IgG and Twist1, IgG and RXR, or IgG and RREB1 ChIP qPCR ΔΔCt values.

In order to examine Twist1 occupancy at shared sequences associated with genes expressed in ECC-Limb or ECC-Limb-PNST groups, ChIP was performed and enrichment at overlapping binding regions was quantified by sequence-specific qPCR. ECC-Limb overlapping binding regions containing E-box binding sites associated with expressed genes from the four major GO categories (Figure 3B) were sorted according to proximity to TSS for greater probability of identifying proximal regulatory sequences, and validation was performed on the two binding peaks nearest the TSS. Intronic binding regions associated with *midline 1 (Mid1)* and *Cdc42 binding protein kinase alpha (Cdc42bpa)* were bound by Twist1 in both ECCs and limb buds (Figure 5A). Of the six binding regions shared among all three-tissue types associated with expressed genes, five binding regions contain E-box consensus sites and four were analyzed by ChIP-qPCR for Twist1 occupancy. Twist1 occupancy at shared binding regions associated with *Chst11, Litaf, Ror2 (Ror-a,* a region 15,965 bp upstream from the TSS*)*, and *Spata5* was confirmed in ECCs, limb buds, and PNST cells, but binding to the shared *CD44* site was not detected (Figure 5B and data not shown). Thus, Twist1 binding was detected at small group of shared binding regions in ECC, limb bud, and PNST cells, which may represent common targets of Twist1 in multiple cell types.

### Identification of potential Twist1 cofactors in ECCs, limb buds, and PNST cells

The presence of tissue-specific Twist1 cofactors was examined as a potential mechanism underlying specificity of target occupancy. Twist1 binding regions associated with expressed genes for each tissue type (Additional files 7, 8 and 9) were analyzed for transcriptional modules containing a transcription factor-binding sequence within +/- 40 base pairs of the E-box consensus site using Genomatix RegionMiner (Additional file 10). In ECC, CP2-erythrocyte factor related to drosophila Elf1 (CP2) sites and heat shock factor sites are located in proximity to E-boxes in approximately 10% of the Twist1 occupied sequences. Retinoid X receptor (RXR) sites (Figure 5E) are present in 43% of the Limb-only Twist1 occupied sites and also in 44% of the ECC-Limb shared Twist1 occupied sites. Interestingly, binding sites for Ras-related element binding protein (RREB1) sites (Figure 5F) are present in 4/5 E-box-containing peaks in ECC-Limb-PNST shared Twist1 binding regions. To validate binding occupancy of Twist1 and candidate cofactors to Twist1 binding regions identified by ChIP-seq, sequence-specific ChIP was performed with antibodies directed against RXR and RREB1 [24, 25]. These analyses confirm that Twist1 and RXR can occupy Twist1 binding regions associated with *Wnt5b* and *Ror2* (*Ror2-a*) in limb buds (Figure 5C). RREB1 binding to shared Twist1 binding region sequences associated with *Spata5, Ror2* (*Ror2-a*)*, Litaf*, and *Chst11* was detected in PNST cells, and RREB1 bound to *Spata5* and *Litaf* Twist1 binding regions in limb buds (Figure 5B,D). However, RREB1 occupancy at shared binding regions was not detected in ECCs. Together these data provide evidence that RXR can also occupy Twist1 binding regions in limb buds, and that RREB1 can occupy Twist1 binding regions in both limb buds and PNST cells, but not ECC. Therefore, RXR is a candidate cofactor for Twist1 in limb buds, and RREB1 is a candidate cofactor for Twist1 in limb buds and PNST cells.

### Twist1 DNA occupancy is associated with genes in the Wnt signaling pathway

**Figure 6:**
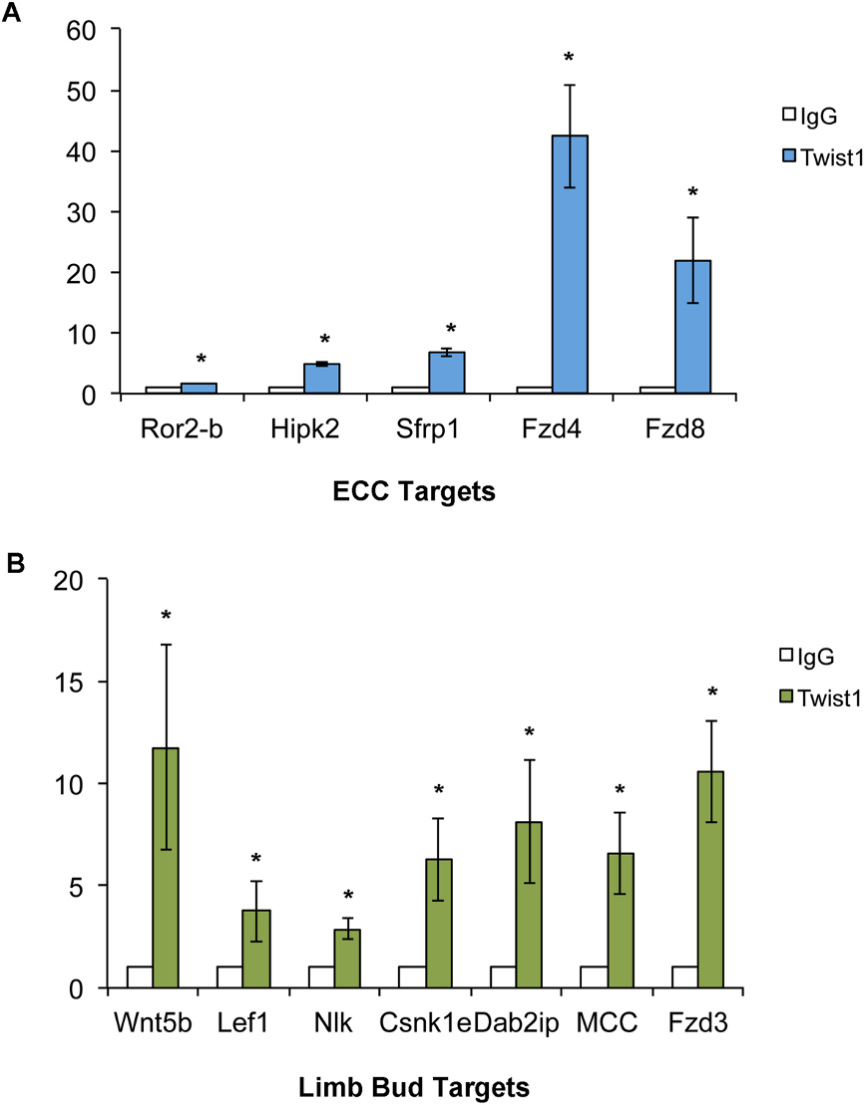
**Twist1 binding region sequences are associated with genes in the Wnt signaling pathway. A**. Wnt pathway genes associated with Twist1 E-box-containing binding regions detected by ChIP-seq in E12.5 ECCs include *receptor tyrosine kinase-like orphan receptor 2 (Ror2-b), homeodomain interacting protein kinase 2 (Hipk2), secreted frizzled-related protein 1 (Sfrp1), frizzled homolog 4 (fzd4),* and *frizzled homolog 8 (fzd8)*. Twist1 binding to these sequences was confirmed by ChIP with anti-Twist1 in E12.5 ECCs followed by quantification by qPCR. **B**. Wnt pathway genes associated with Twist1 E-box containing binding regions detected by ChIP-seq in E10.5 limb buds include *Wnt5b, lymphoid enhancer binding factor 1 (Lef1), nemo like kinase (Nlk), casein kinase 1, epsilon (Csnk1e), disabled 2 interacting protein (Dab2ip), mutated in colorectal cancers (Mcc)*, and *frizzled homolog 3 (Fzd3).* Twist1 binding to these sequences was confirmed by ChIP with anti-Twist1 in E10.5 limb bud followed by quantification by qPCR. All ChIP experiments were quantified with qPCR, and fold-enrichment was calculated relative to normal rabbit IgG control. All analyses were performed in biological triplicate. * indicates p < 0.05 as determined by student’s *t*-test comparing IgG and Twist1 ChIP qPCR ΔΔCt values.

To examine pathways corresponding to genes associated with Twist1 binding regions, ToppGene Suite and GeneMANIA were utilized to integrate information on protein-protein interactions, gene regulation, coexpression, and functional studies, as well as to determine pathway association [26]. These analyses demonstrated that genes associated with Wnt signaling pathway components and interacting proteins are prominent in binding regions within 50 Kb of a TSS associated with expressed genes from ECC-Limb shared Twist1 binding peaks. In ECCs, limb buds, and PNST cells, Twist1 occupied a binding peak (*Ror2-a)* associated with the Wnt receptor *Ror2* (Figure 5B). Twist1 binding to peak sequences associated with Wnt pathway genes was confirmed by ChIP-qPCR in E12.5 ECCs and E10.5 limb buds. In ECCs, Twist1 bound sequences associated with a second region associated with *Ror2 (Ror2-b,* located 4,444 bp from the TSS*),* as well as sequences associated with *homeodomain interacting protein kinase 2 (Hipk2), secreted frizzled-related protein 1 (Sfrp1), frizzled homolog 4 (fzd4),* and *frizzled homolog 8 (fzd8)* (Figure 6A). In limb buds, Twist1 binding to peak sequences associated with *wingless-related MMTV integration site 5B (Wnt5b), lymphoid enhancer binding factor 1 (Lef1), nemo like kinase (Nlk), casein kinase 1, epsilon (Csnk1e), disabled 2 interacting protein (Dab2ip), mutated in colorectal cancers (Mcc)*, and *frizzled homolog 3 (Fzd3)* were confirmed (Figure 6B). Thus Twist1 binding was detected at genomic sequences associated with Wnt pathway genes and Wnt interacting genes in developing ECCs and limb buds.

## Discussion

Tissue-specificity of Twist1 binding to distinct subsets of gene sequences was observed by ChIP-seq and then confirmed by ChIP and sequence-specific qPCR in ECC, limb buds, and PNST cells. The tissue-specific binding regions occupied by Twist1 are consistent with known functions of Twist1 in each cell type, although the individual genes are distinct. Twist1 binds to DNA sequences associated with genes that regulate migration and other biological functions in each tissue, but few shared binding regions were detected between samples. Among the six sequences occupied by Twist1 in all three tissues are genes involved in cell adhesion-migration that are also bound by the Ras-related element binding protein (RREB1). Moreover, Twist1 binding regions are associated with several Wnt pathway genes and pathway components. These data suggest that Twist1 binds target genomic sequences predominantly in a tissue-specific manner and provide initial evidence that Twist1 occupies sequences of genes acting in the Wnt signaling pathway. However, the mechanisms that dictate the tissue-specificity of Twist1 binding to distinct transcriptional targets in different cell types remain to be determined.

Analysis of Twist1 and its downstream target genes in a variety of cell types have revealed roles in cell proliferation, adhesion/migration, and ECM protein expression. In the ECC mesenchymal cells of the developing heart valves, alterations in Twist1 expression affect cell migration and expression of *Tbx20, Cadherin-11*, and *Periostin* [2, 9]. Additionally, Twist1 binding to genomic regions associated with genes that regulate cell proliferation and migration, including *Tbx20, Cdh11, Rab39b, Sema3C,* and *Gadd45a,* have been identified [5]. Likewise, expression analysis of AVC of Twist1-null embryos relative to WT mice revealed reduced expression of cell migration and ECM genes [12]. Interestingly, *Ror2* expression is decreased in Twist1-null ECC and also was found to contain a Twist1 binding region in this study [12]. In synovial sarcoma cells, Twist1 direct downstream targets are involved in maintenance of mesenchymal differentiation [23], and Twist1 targets promote cell migration and proliferation in thyroid cancer cells [27]. Interestingly, in the current study only one binding region, *Ror2*, overlaps with these 3 other Twist1 ChIP-seq studies. These studies further support the ability of Twist1 to regulate different networks of downstream genes depending on the specific cellular context. However, predominant roles for Twist1 downstream target genes in cell migration and adhesion are highly reproducible.

Potential Twist1 cofactors were identified based on the presence of transcription factor binding sites in Twist1 binding regions and were confirmed by ChIP-qPCR in the tissues of interest. The retinoic acid (RA) receptor RXR was identified as a potential Twist1 cofactor in limb buds. Both RA signaling and Twist1 have been implicated in limb bud patterning, and the binding of both factors to the same genomic target sequence supports RXR as a candidate cofactor for Twist1 [4, 28]. In skeletal muscle, the bHLH transcription factor MyoD physically interacts with RXR to promote cell-type specific gene regulation, suggesting the possibility of a related or intersecting regulatory mechanism with Twist1 [24]. RREB1 binding sites are present in proximity to Twist1 occupied sites detected in ECC, limb buds, and PNST cells. RREB1 is expressed in developing tissues and cancer where it acts downstream of Ras and MAPK signaling Twist1 is required for K-Ras mediated progression of lung tumors, supporting a role for RREB1 in this regulatory interaction [29, 30]. In addition, RREB1 has been shown to interact and bind with bHLH factor NeuroD to enhance transcriptional activity [25, 30]. Thus Twist1 and RREB1 may act together in transcriptional regulation of genes activated by Ras-MAPK signaling in multiple tissue types. Together, these studies have uncovered candidate cofactors, previously demonstrated to interact with related bHLH factors, which may contribute to the cell-type specificity of Twist1 genomic occupancy and target gene regulation.

Increasing evidence implicates the Wnt signaling pathway as a key pathway active in developing heart valves and limb buds and also in disease states, such as cancer [31]. Twist1 genomic occupancy was observed in association with genes in the Wnt pathway in ECC and limb buds. In ECCs, Wnt/β-catenin signaling is required for EMT of the endothelial cells, and for proliferation of mesenchymal cells [32, 33]. In Twist1-null AVC and OFT, genes associated with the Wnt signaling pathway are significantly differentially regulated relative to control embryos [12]. Likewise, conditional deletion of the Twist1 target gene *Tbx20* in endothelial cells leads to reduced expression of Wnt pathway genes *Sfrp1* and *Fzd4* in ECCs [34]. The current study identified genomic sequences associated with *Sfrp1* and *Fzd4* occupied by Twist1 in ECCs, supporting an intersecting regulatory network for Twist1, Tbx20, and Wnt signaling in heart valve development. In developing limb buds, Wnt signaling is critical for limb bud outgrowth, while Wnt and RA signaling are required for proximal-distal limb patterning [35–37]. Here, we identify *Wnt5b* associated sequences bound by Twist1 and RXR in limb buds. In limb bud cartilage progenitors, RA and Wnt signaling pathways intersect, and Wnt5b promotes cell migration and inhibition of cell adhesion mediated by cadherins, which are often regulated by Twist1 [38, 39]. Additionally, mutations in Wnt/β-catenin signaling pathway genes have been identified in patients with neurofibromatosis type 1, and in vitro experiments in MPNST cells demonstrate that Wnt signaling promotes cell proliferation [40, 41]. Furthermore, Wnt signaling through Ror2 is linked with cell invasion and EMT in human osteosarcoma cells, and the same binding region, *Ror2-b*, was recently identified as a Twist1 target in both E10.5 AVC of the heart and developing limb buds [12, 42]. The identification of Twist1 binding regions in proximity to multiple genes associated with Wnt signaling suggests that Twist1 may regulate components of the Wnt signaling pathway in ECCs, limb buds, and PNST cells.

## Conclusions

This study examined Twist1 genomic occupancy in two murine developmental tissues (E12.5 ECCs and E10.5 limb buds) and a murine cancer cell line (PNST cells). Interestingly, little overlap between binding regions was observed in ECCs, limb buds, and PNST cells, although the predominant functions of Twist1 occupied genes, including cell adhesion and migration are similar in the three tissues. The specificity of Twist1 binding was confirmed for tissue-specific targets, while Twist1 binding in multiple tissues was detected for shared target genes, including four genes that represent core targets of Twist1 in multiple cell types. The molecular basis for the tissue-specificity of Twist1 binding remains to be determined. Interestingly RREB1 binding sites are located in proximity to Twist1 sites in the occupied regions detected in multiple tissues providing initial evidence for a core regulatory interaction. In addition, we provide evidence that Twist1 interacts with genes in the Wnt signaling pathway in ECCs, limb buds, and PNST cells. A limitation of our analysis is the focus on Twist1 genomic occupancy of expressed genes, which is indicative of Twist1 acting as a transcriptional activator, not repressor. However, the identification of tissue-specific targets and DNA binding mechanisms of genes potentially activated by Twist1 will aid in our understanding of its role in transcriptional hierarchies active in development and disease.

## Methods

### Ethics statement

All experiments involving animals were performed using experimental protocols and procedures reviewed and approved by the Cincinnati Children’s Hospital Medical Center Biosafety Committee and Institutional Animal Care and Use Committee.

### Chromatin immunoprecipitation (ChIP) sample isolation

FVBN (Taconic) wild-type mouse litters were generated from timed matings, where presence of the copulation plug was considered E0.5. Atrioventricular canal (AVC) ECC tissue from E12.5 embryos was dissected in 1X phosphate buffered saline (1XPBS) with tungsten needles and pooled from 10-12 individuals for each ChIP experiment [43, 44]. Forelimb bud tissue from 10-12 E10.5 embryos was isolated and pooled in 1XPBS. Mouse peripheral nerve sheath tumor (PNST) cells were cultured in DMEM medium (Gibco) containing 10% fetal bovine serum (FBS, Hyclone) and 1% penicillin-streptomycin (pen-strep, Invitrogen) [15, 45]. PNST cells were cultured in 100mm tissue culture plates, and cells from 1 plate were utilized for each experiment when they reached 80-90% confluency. Genomic DNA from ECCs, limb buds, and PNST cells was cross-linked and isolated according to manufacturer’s protocols (Magnify ChIP kit, Invitrogen). Crosslinked DNA complexes were sheared by sonication (Virsonic 60, Virtis, output of 5) 6 times for 5 seconds with a 2-minute refractory period on ice. Samples were sheared to a size of 150-250 basepairs (bp). ChIP was then performed according to the manufacturer’s protocol (Magnify ChIP Kit, Invitrogen). Immunoprecipitation was performed with anti-Twist1 (Sigma, T6451, 5 μg) [5, 33], anti-RREB1 (Santa Cruz, sc-138055, 5 μg) [25], and anti-RXR (Santa Cruz, sc-774, 5 μg) [46] or with the appropriate IgG control (Magnify ChIP Kit, Invitrogen, 492024, 5 μg).

### ChIP-seq

Approximately 5 litters of FVB/N AVC ECCS and limb buds and 2, 100 mm culture plates of PNST cells were isolated and pooled for ChIP-seq analysis. Each ChIP-seq sample yielded approximately 10 ng of DNA for sequencing with Illumina HiSeq2000. A single read genomic DNA library was generated using an IntegenX robot (Apollo 324) and PrepX DNA library kit (IntegenX 400040). NextGen high-throughput sequencing was performed on ChIP DNA fragments using an Illumina HiSeq2000 with 10 million raw reads per sample following the manufacturer’s protocols. Peaks were called using Model-based Analysis of ChIP-seq (MACS, v1.4.2) using the following parameters, band width = 300, and p-value cut off of 1.00e-05 relative to a whole mouse genome data set generated from wild type FVB/N mouse genomic DNA as a control. 50bp sequence reads were mapped to the mouse genome (NCBI Build 37, mm9) with Illumina gapped aligner Eland v2 and Bowtie to identify the nearest transcriptional start site upstream and downstream of the peak [47]. ChIP-seq datasets of unaligned reads and reads aligned to the genome for E12.5 ECCs, E10.5 limb buds, and PNST cells are available on the Gene Expression Omnibus (GEO, accession #GSE50111).

### *de-novo*motif analysis

In order confirm that E-box binding consensus sequences were enriched throughout the ChIP-seq datasets for E12.5 ECCs, E10.5 limb buds, and PNST cells *de-novo* motif analysis was performed using PscanChIP, which utilizes the TRANSFAC database, and MEME-ChIP, which utilizes the JASPAR database [18, 19]. Chromosomal coordinates for binding peaks following MACS analysis were used as input for PscanChIP analysis, and sequences of binding peaks following MACS analysis were used as input for MEME-ChIP.

### Gene expression data

Mouse gene expression datasets generated by Mouse Genome 430 2.0 Array (Affymetrix) and raw data files (.cel) were obtained from the GEO database for developing heart valves (E12.5 ECCs and E17.5 valves [GSE11040]), and developing forelimb buds (E10.5 forelimb buds and E13.5 forelimb buds [GSE30138]) [10, 20]. Raw Affymetrix microarray expression data (.cel) files were processed with GeneSpring v12.1 software with robust multichip average (RMA) analysis [5, 10]. Pairwise analysis was performed on datasets from the same GEO submission, E12.5 ECCs versus E17.5 valves, E10.5 forelimbs versus E13.5 forelimbs. Developmentally expressed genes were identified as genes present in E12.5 ECCs (≥1 fold expression in E12.5 ECCs v. E17.5 limb buds) or in E10.5 limb buds (≥1 fold expression in E10.5 limb buds v. E13.5 limb buds). These lists of probe sets associated with genes include genes expressed at both developmental stages or preferentially at the earlier stage, but do not include the genes expressed preferentially at later stages of valve or limb maturation. Genes enriched in human MPNST and mouse PNST cells, as compared to normal nerves for each species, were identified as described [14, 16]. These probe set expression lists were used for comparison to genomic sequences bound by Twist1, as determined by ChIP-seq.

Genes with Twist1 ChIP-seq peaks located within 50 Kb of the transcriptional start site (TSS) were compared to E12.5 ECC, E10.5 limb bud, and human MPNST/mouse PNST gene expression microarray lists using GeneSpring v12.1 software (Agilent). Genes associated with Twist1 ChIP-seq peaks for ECC, limb bud, and PNST cells within 50 Kb of a TSS were imported into GeneSpring for conversion of each gene into corresponding probe sets on the Mouse Genome 430 2.0 (Affymetrix) Array platform. Probe sets from ChIP-seq peak associated gene lists and corresponding tissue-specific probe set expression lists (ECCs, limb buds, and shared human MPNST/mouse PNST cells) were compared. Overlapping expression was represented in Venn diagrams and shared probe sets were identified. The genes associated with shared ChIP-seq probe sets and tissue-specific expressed probe sets were analyzed for Gene Ontology (GO) enrichment using ToppGene and PANTHER databases [26, 48].

### Analysis of ChIP-seq datasets

ECC, limb bud, and PNST Twist1 ChIP-seq peaks associated with genes that are expressed in each cell type were then compared with each other to identify peaks shared between datasets (ECC-Limb, ECC-PNST, Limb-PNST, and ECC-Limb-PNST) using IntersectBed from Nebula with a minimum overlap of 5% of the nucleotides in shared peaks [21]. Genomic sequences for each of the binding peak sequences (~50 bp to 750 bp in length) were obtained using Genomatix Software Suite v2.7. RegionMiner (Genomatix Software Suite v2.7, Release 4.7) was used to determine the enriched transcription factor-binding sites (TFBS) present in each data set. Peaks associated with expressed genes in ECC only, limb bud only, PNST only, ECC-Limb shared, ECC-PNST shared, and sequences shared in the three cell types (ECC-Limb-PNST) were extended +/- 200 bp. Transcription modules containing an E-box binding site plus another binding site with a z-score ≤ -2.0 within 40 bp of an E-box consensus site were identified. Lists of genes associated with Twist1 binding peaks unique to each cell type or shared among multiple cell types were examined for pathway identification and interactions using ToppGene Suite and GeneMANIA (http://www.genemania.org) [26, 49].

### Quantitative real-time PCR (qPCR) analysis of ChIP

Relative abundance of specific genomic sequences in eluted DNA from ChIP experiments was quantified by qPCR compared to normal rabbit IgG control as previously described [5]. qPCR amplification began with an initial denaturation step of 94°C for 3 minutes followed by 40 cycles of 94°C for 20 sec, annealing step for 30 seconds (annealing temperature was 55°C for all primer sets), 72°C for 30 seconds. Fold-enrichment was calculated relative to IgG control, which was set to 1.0. Primer sequences, distance of the binding peak from the nearest transcriptional start site, and annealing temperatures are listed in Additional file 11.

### Availability of supporting data

The data sets supporting the results of this article are available in the GEO database, [GSE50111].

## Electronic supplementary material

Additional file 1: **Table of E12.5 ECC Twist1 ChIP-seq peak determination by MACS analysis.** Twist1 ChIP-seq was performed in E12.5 ECCs and sequencing results were analyzed by MACS version 1.4.2 with default settings. The table contains the peak chromosomal location (chromosome (chr), start, and end), length of peak, summit of peak, number of tags at peak location, p value, fold enrichment, distance from nearest downstream gene and gene name, distance from nearest upstream gene and gene name. (XLS 2 MB)

Additional file 2: **Table of E10.5 limb bud Twist1 ChIP-seq peak determination by MACS analysis.** Twist1 ChIP-seq was performed in E10.5 forelimb buds and sequencing results were analyzed by MACS version 1.4.2 with default settings. The table contains the peak chromosomal location (chromosome (chr), start, and end), length of peak, summit of peak, number of tags at peak location, p value, fold enrichment, distance from nearest downstream gene and gene name, distance from nearest upstream gene and gene name. (XLS 1 MB)

Additional file 3: **Table of peripheral nerve sheath tumor (PNST) cell Twist1 ChIP-seq peak determination by MACS analysis.** Twist1 ChIP-seq was performed in PNST cells and sequencing results were analyzed by MACS version 1.4.2 with default settings. Table contains the peak chromosomal location (chromosome (chr), start, and end), length of peak, summit of peak, number of tags at binding peak location, p value, fold enrichment, distance from nearest downstream gene and gene name, distance from nearest upstream gene and gene name. (XLS 1 MB)

Additional file 4: **E-box containing motifs are significantly enriched in Twist1 ChIP-seq peaks for E12.5 ECCs, E10.5 limb buds, and PNST cells.** PscanChIP analysis E12.5 ECCs, and and MEME-ChIP analysis was performed on peaks for E10.5 limb buds, and PNST cells following MACS analysis. The position weight matrix of the most enriched E-box containing motif (underlined) with associated p-values in E12.5 ECCs and PNST cells and the e-value associated with E10.5 limb buds are represented. (TIFF 1 MB)

Additional file 5: **Overlap of genes associated with Twist1 ChIP-seq peaks and genes expressed E12.5 ECCs, E10.5 limb buds, or PNST cells.** Venn diagrams are shown for A. Gene probe sets corresponding to genes expressed in E12.5 ECCs versus genes associated with binding regions within 50 Kb of a TSS from Twist1 ChIP-seq in E12.5 ECCs. B. Gene probe sets corresponding to genes expressed in E10.5 limb buds versus genes associated with binding regions within 50 Kb of a TSS from Twist1 ChIP-seq in E10.5 limb buds. C. Gene probe sets corresponding to genes shared between mouse PNST cells and human MPNST cells versus genes associated with binding regions within 50 Kb of a TSS from Twist1 ChIP-seq in PNST cells. Note that multiple gene probe sets are present for many genes in the microarray analysis. (TIFF 2 MB)

Additional file 6: **The locations of Twist1 binding regions detected by ChIP-seq are typical for transcription factor binding.** A. The genomic locations of Twist1 binding regions detected by ChIP-seq were analyzed relative to the nearest transcriptional start site using Genomatix RegionMiner. ChIP-seq peak location analysis was performed for cell-type specific peaks (ECC only, limb bud only, and PNST only ChIP-seq peaks within 50 Kb of nearest TSS and expressed in the tissue of interest) and shared peaks (ECC-limb bud, ECC-PNST, and Limb-PNST that have E-box consensus sites). (TIFF 809 KB)

Additional file : **Table of gene lists for the four most predominant gene ontology (GO) categories associated with Twist1 binding regions as determined by ChIP-seq in E12.5 ECCs.** The name and description of genes in the four gene ontology categories, cell-cell signaling, cell adhesion, cell morphogenesis, and neurogenesis, from the expressed genes associated with Twist1 binding regions within 50 Kb of a TSS in E12.5 ECCs. The presence of an E-box was not verified in this list. GO analysis was performed using ToppGene. (XLSX 59 KB)

Additional file 8: **Table of gene lists for the four most predominant gene ontology (GO) categories associated with Twist1 binding regions as determined by ChIP-seq in E10.5 limb buds.** The name and description of genes in the four GO categories, kinase activity, cell migration, neurogenesis, and cytoskeletal proteins, from the expressed genes associated with binding regions within 50 Kb of a TSS in E10.5 limb buds. The presence of an E-box was not verified in this list. GO analysis was performed using ToppGene. (XLSX 55 KB)

Additional file 9: **Table of gene lists for the four most predominant gene ontology (GO) categories associated with Twist1 binding regions as determined by ChIP-seq in PNST cells.** The name and description of genes in the four GO categories, cell adhesion, skeletal system development, cell recognition, and ventricular septum morphogenesis, from the expressed genes associated with binding regions within 50 Kb of a TSS in PNST cells. The presence of an E-box was not verified in this list. GO analysis was performed using ToppGene. (XLSX 42 KB)

Additional file 10: **Twist1 ChIP-seq binding regions have distinct candidate cofactor consensus binding sites in ECC, limb buds and PNST cells.** Twist1 ChIP-seq binding regions associated with genes from the top four gene ontology (GO) categories for E12.5 ECCs, E10.5 limb buds, or PNST cells (Figure 2) were analyzed for the presence of an E-box adjacent to another transcription factor-binding site within 40 base pairs, defined as a module, using Genomatix RegionMiner. The table includes the module, number of peak sequences that contain the module, and module family information. Each cell type has different predicted transcription factor binding sites within close proximity to E-box consensus sites. (TIFF 2 MB)

Additional file 11: **Table of Sequences of the primers used for ChIP-seq validation.** Sequences of primers used for sequence-specific qPCR following ChIP with anti-Twist1 and distance of corresponding peak from the gene TSS are shown. (XLS 26 KB)
