# Supplementary figures and images for: Genome-wide Twist1 occupancy in endocardial cushion cells, embryonic limb buds, and peripheral nerve sheath tumor cells
Source: BMC Genomics. 2014 Sep 28;15(1):821. doi: 10.1186/1471-2164-15-821 (PMC4190347; doi:10.1186/1471-2164-15-821)

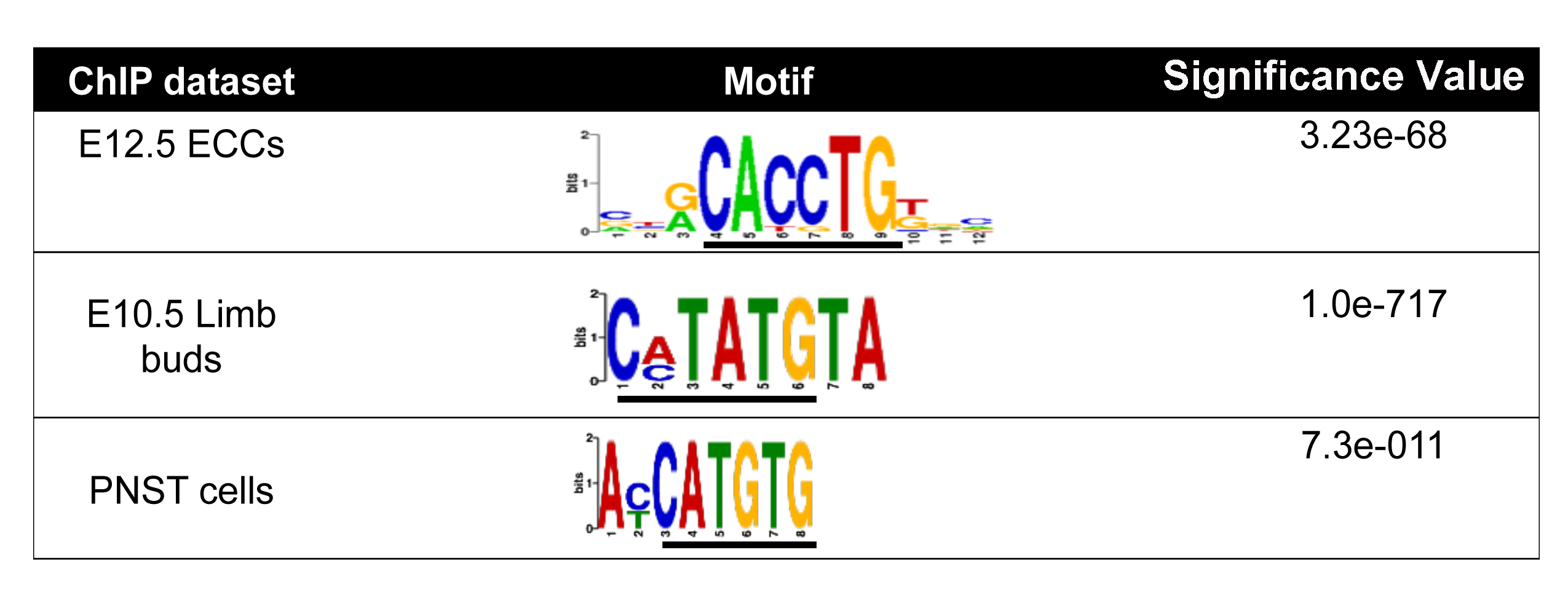

Supplement: Supplementary file 4 — Additional file 4: E-box containing motifs are significantly enriched in Twist1 ChIP-seq peaks for E12.5 ECCs, E10.5 limb buds, and PNST cells. PscanChIP analysis E12.5 ECCs, and and MEME-ChIP analysis was performed on peaks for E10.5 limb buds, and PNST cells following MACS analysis. The position weight matrix of the most enriched E-box containing motif (underlined) with associated p-values in E12.5 ECCs and PNST cells and the e-value associated with E10.5 limb buds are represented. (TIFF 1 MB) [file 12864_2014_6501_MOESM4_ESM.tiff]

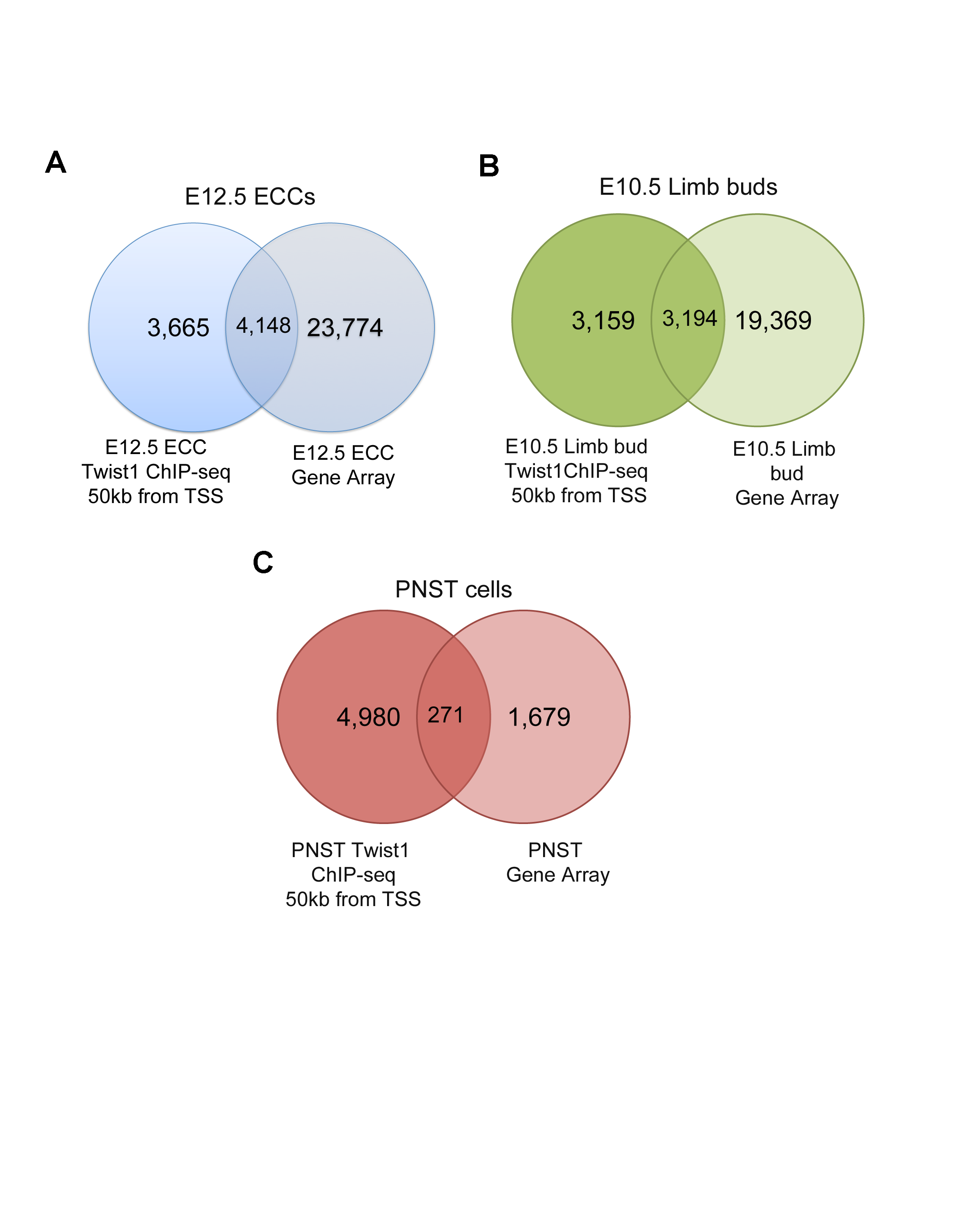

Supplement: Supplementary file 5 — Additional file 5: Overlap of genes associated with Twist1 ChIP-seq peaks and genes expressed E12.5 ECCs, E10.5 limb buds, or PNST cells. Venn diagrams are shown for A. Gene probe sets corresponding to genes expressed in E12.5 ECCs versus genes associated with binding regions within 50 Kb of a TSS from Twist1 ChIP-seq in E12.5 ECCs. B. Gene probe sets corresponding to genes expressed in E10.5 limb buds versus genes associated with binding regions within 50 Kb of a TSS from Twist1 ChIP-seq in E10.5 limb buds. C. Gene probe sets corresponding to genes shared between mouse PNST cells and human MPNST cells versus genes associated with binding regions within 50 Kb of a TSS from Twist1 ChIP-seq in PNST cells. Note that multiple gene probe sets are present for many genes in the microarray analysis. (TIFF 2 MB) [file 12864_2014_6501_MOESM5_ESM.tiff]

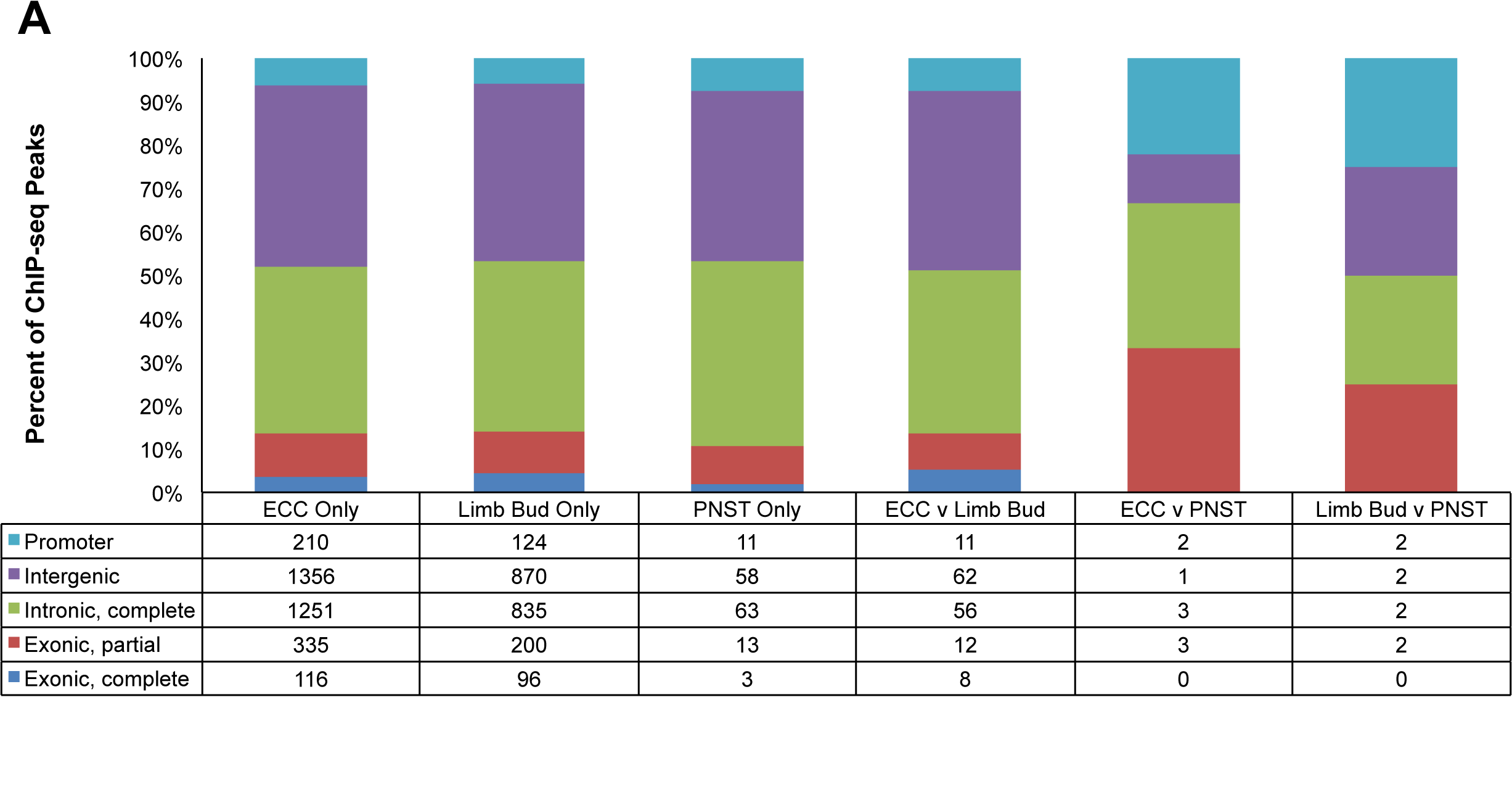

Supplement: Supplementary file 6 — Additional file 6: The locations of Twist1 binding regions detected by ChIP-seq are typical for transcription factor binding. A. The genomic locations of Twist1 binding regions detected by ChIP-seq were analyzed relative to the nearest transcriptional start site using Genomatix RegionMiner. ChIP-seq peak location analysis was performed for cell-type specific peaks (ECC only, limb bud only, and PNST only ChIP-seq peaks within 50 Kb of nearest TSS and expressed in the tissue of interest) and shared peaks (ECC-limb bud, ECC-PNST, and Limb-PNST that have E-box consensus sites). (TIFF 809 KB) [file 12864_2014_6501_MOESM6_ESM.tiff]

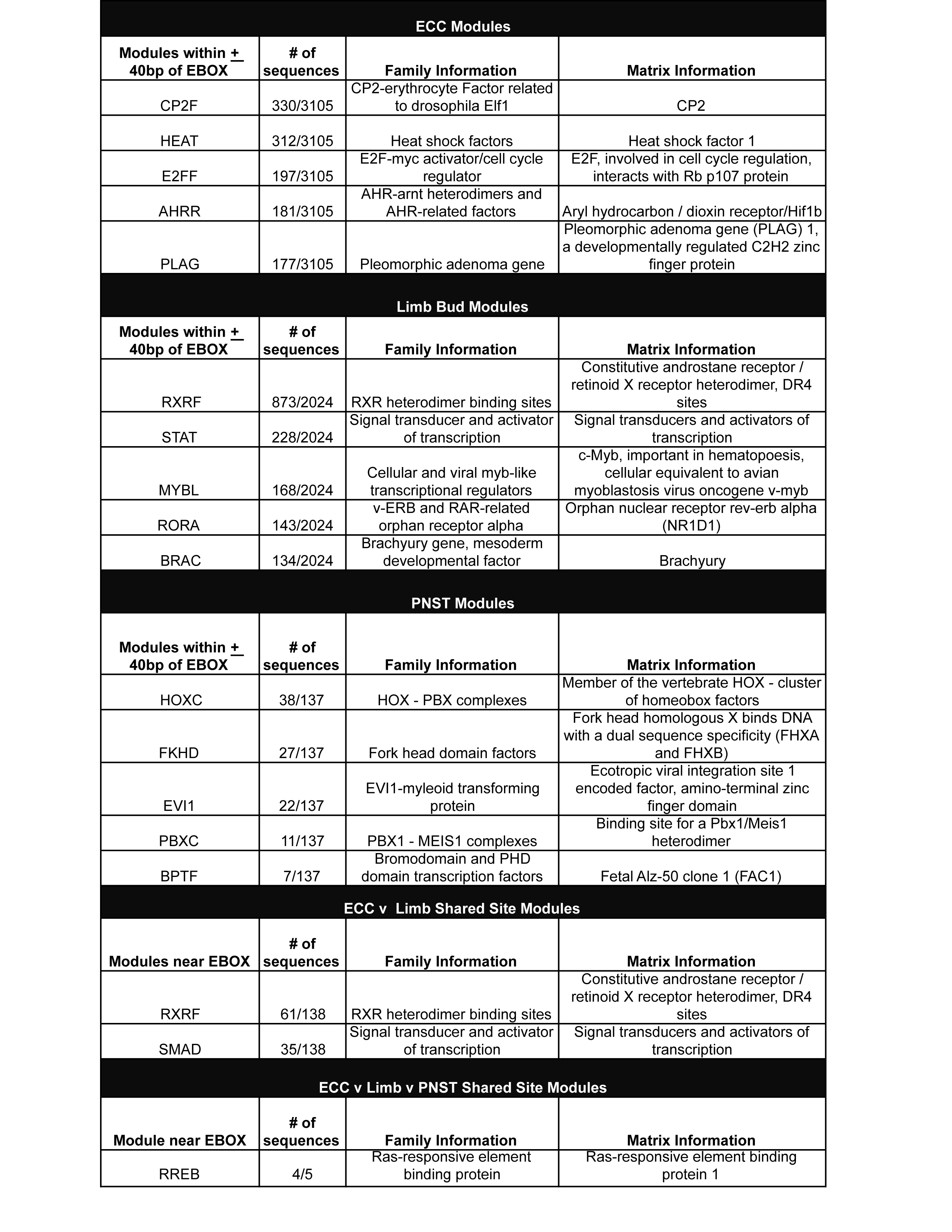

Supplement: Supplementary file 10 — Additional file 10: Twist1 ChIP-seq binding regions have distinct candidate cofactor consensus binding sites in ECC, limb buds and PNST cells. Twist1 ChIP-seq binding regions associated with genes from the top four gene ontology (GO) categories for E12.5 ECCs, E10.5 limb buds, or PNST cells (Figure 2) were analyzed for the presence of an E-box adjacent to another transcription factor-binding site within 40 base pairs, defined as a module, using Genomatix RegionMiner. The table includes the module, number of peak sequences that contain the module, and module family information. Each cell type has different predicted transcription factor binding sites within close proximity to E-box consensus sites. (TIFF 2 MB) [file 12864_2014_6501_MOESM10_ESM.tiff]
